# Radiofrequency Ablation for Hepatocellular Carcinoma Adjacent to the Bile Duct Via Intraductal Cooling Through an Endoscopic Nasobiliary Drainage Tube

**DOI:** 10.14309/crj.0000000000000343

**Published:** 2020-03-16

**Authors:** Yusuke Seiki, Satoshi Tanaka, Seiya Kato, Akio Ishihara, Shoichi Nakazuru, Hisashi Ishida, Eiji Mita

**Affiliations:** 1 Department of Gastroenterology and Hepatology, National Hospital Organization Osaka National Hospital, Osaka, Japan

## CASE REPORT

**Figure 1.**
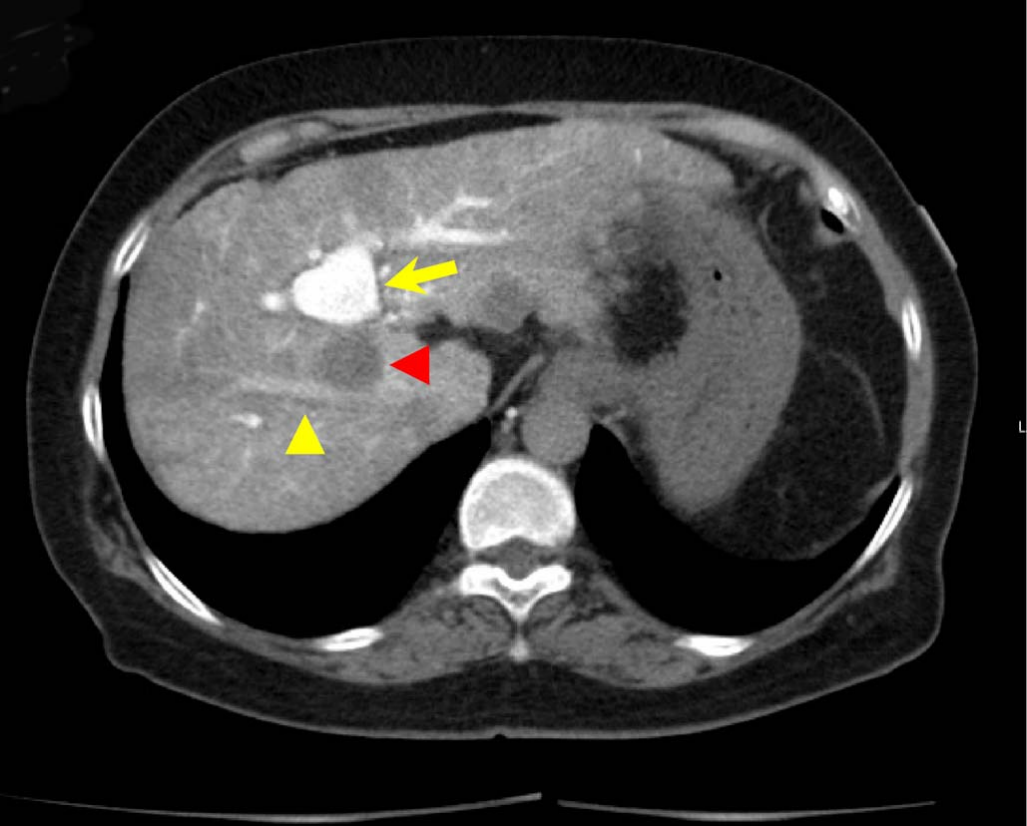
Computed tomography scan showing the hepatocellular carcinoma nodule (red arrowhead) adjacent to the intrahepatic B8 bile duct (yellow arrowhead). The high-density lesion observed above the hepatocellular carcinoma nodule (yellow arrow) is the transcatheter arterial chemoembolization scar.

**Figure 2.**
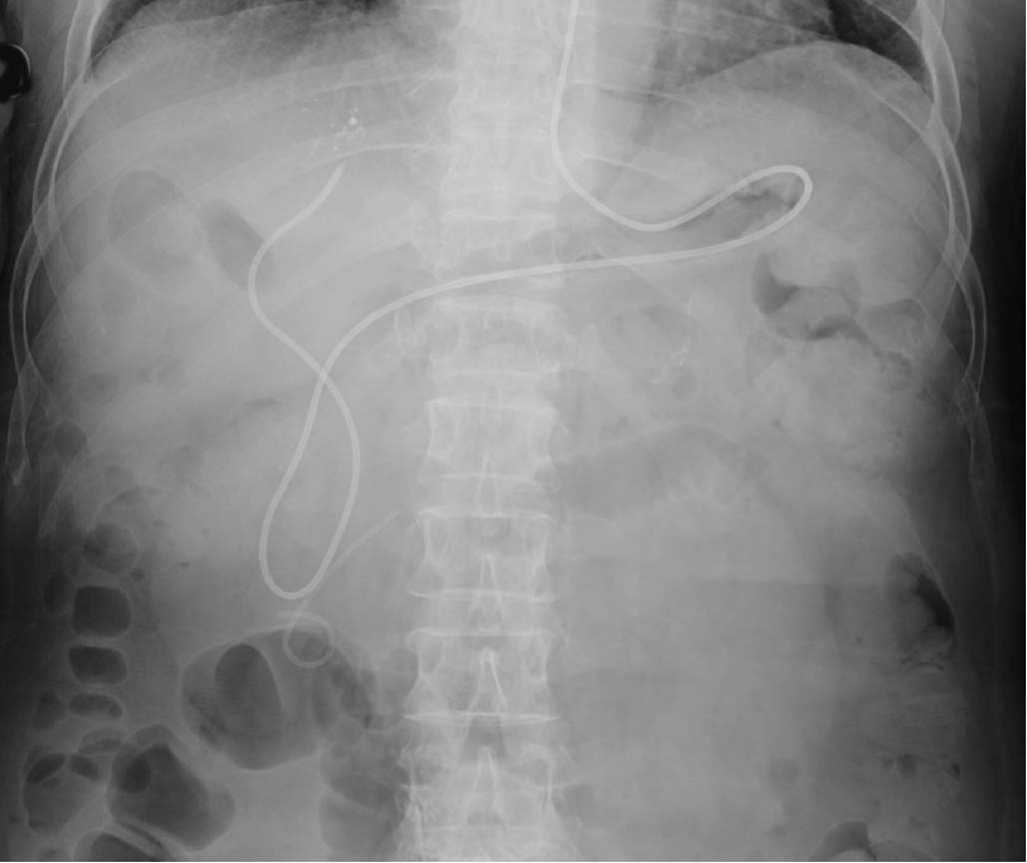
An endoscopic nasobiliary drainage tube has been placed.

**Figure 3.**
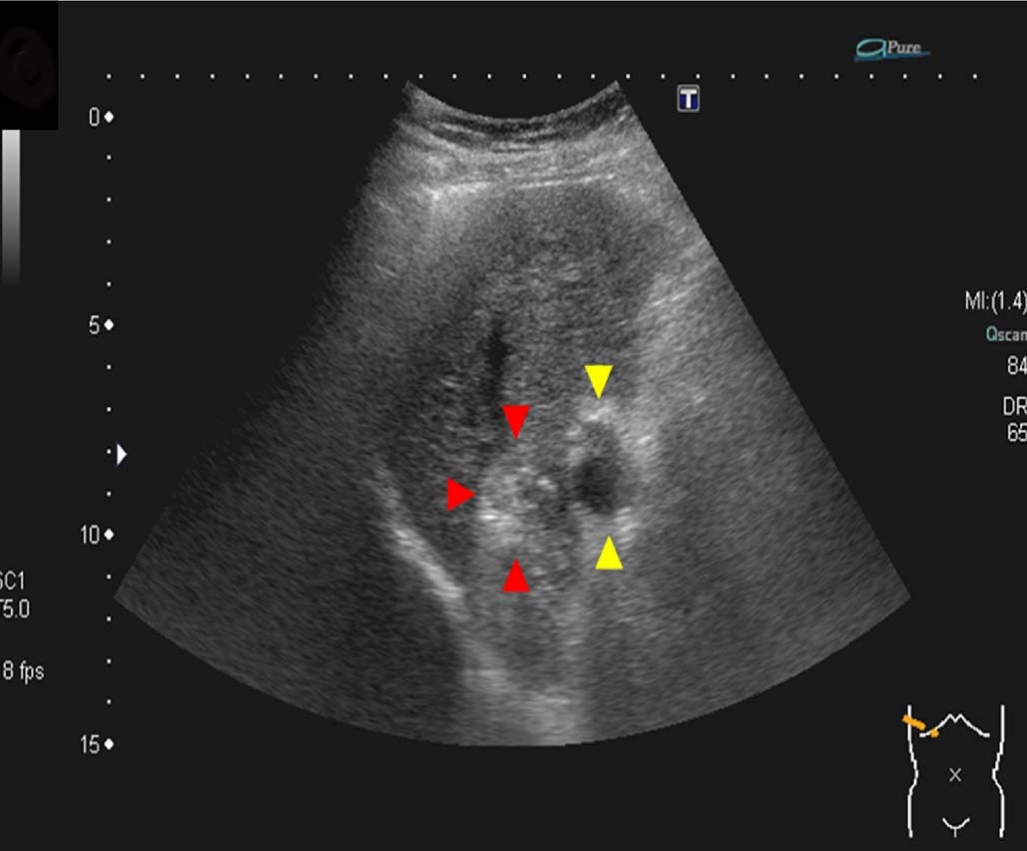
Ultrasound confirming enhancement of the bile duct (yellow arrowheads) adjacent to the hepatocellular carcinoma nodule (red arrowheads).

**Figure 4.**
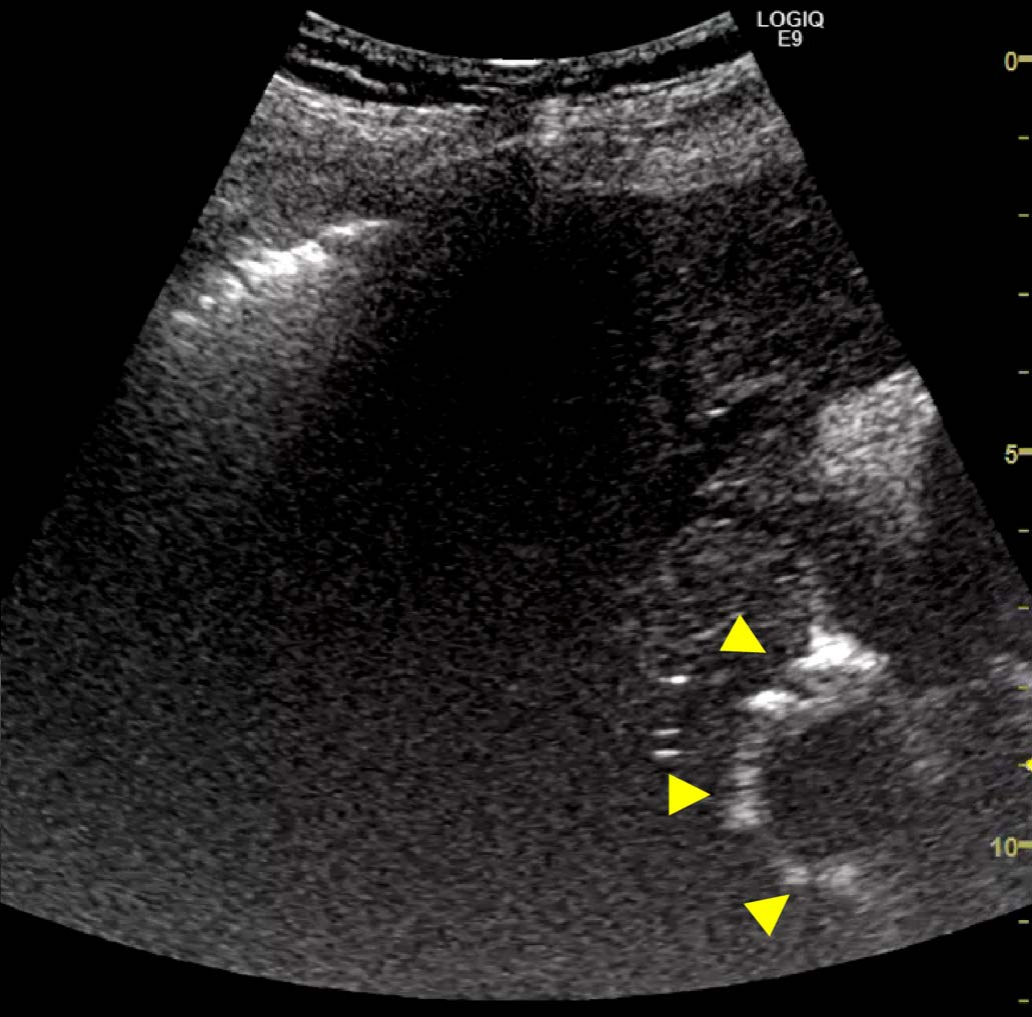
Ultrasound confirming enhancement of the peripheral bile duct close to the lesion with no bile duct injury (yellow arrowheads).

**Figure 5.**
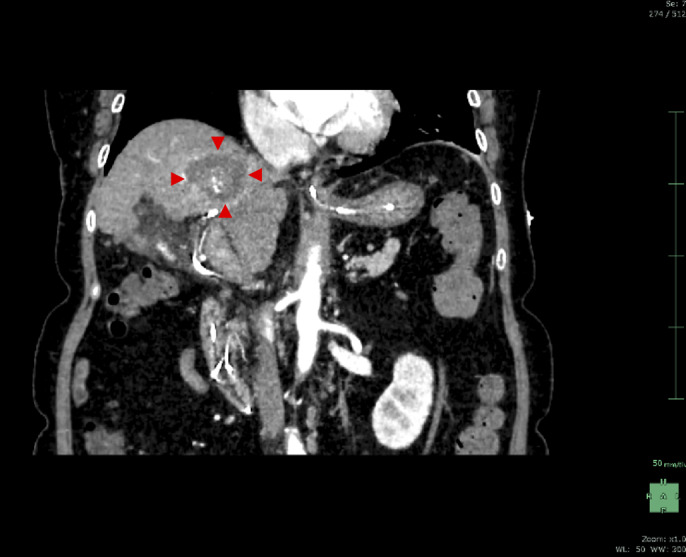
Follow-up computed tomography scan showing complete ablation of the hepatocellular carcinoma (red arrowheads).

A 68-year-old woman underwent radiofrequency ablation (RFA) for hepatocellular carcinoma (HCC) in segment 8 after transcatheter arterial chemoembolization. Pretreatment computed tomography scans revealed that the HCC nodule was adjacent to the intrahepatic B8 bile duct (Figure 1). A 6-Fr endoscopic nasobiliary drainage (ENBD) tube was inserted into the B8 bile duct 1 day before RFA (Figure 2). Before RFA, a perflubutane-based contrast agent (Sonazoid, Daiichi Sankyo, Tokyo, Japan) was injected through the ENBD tube to confirm enhancement of the bile duct adjacent to the HCC nodule (Figure 3). Ultrasonography-guided RFA was performed for the 26 × 33-mm lesion in segment 8 and saline chilled to 4°C was infused into the bile duct through the ENBD tube at a rate of 60 mL/min. Finally, the contrast agent was reinjected through the ENBD tube to confirm enhancement of the peripheral bile duct close to the lesion and absence of bile duct injury (Figure 4). Contrast-enhanced computed tomography scans obtained the day after RFA revealed complete ablation of the HCC lesion with no signs of bile duct injury (Figure 5).

**Video 1:** Radiofrequency ablation (RFA) procedure with bile duct cooling. (Watch the video at http://links.lww.com/ACGCR/A19.)

RFA is a minimally invasive treatment for HCC, with high safety and efficacy. However, several complications have been reported; bile duct injury is among the most severe. It is caused by thermal damage during ablation, and its occurrence mostly depends on the distance between the targeted tumor and the intrahepatic bile duct.^1^ Therefore, when the target lesion is close to the bile duct, intraductal cooling with an ENBD tube is useful for preventing bile duct injury.^2^ However, heat loss on cooling the bile duct, known as the “heat-sink effect,” may lead to incomplete ablation.^3^ Although the ablation time usually lasts less than 12 minutes, we extended it to 19 minutes for this treatment. The patient has been carefully followed for 1 year, with no evidence of bile duct injury or local recurrence till date.

Contrast-enhanced ultrasound cholangiography is safe and useful for real-time visualization of the bile ducts during hepatobiliary surgery.^4^ We, therefore, used this method for evaluating bile duct injury during RFA. This is the first video report on contrast-enhanced ultrasound cholangiography. We present a case of HCC near the bile duct in which RFA was successfully performed with intraductal chilled saline perfusion via an ENBD tube; contrast agent injection through the ENBD tube was useful for evaluating bile duct injury.

## DISCLOSURES

Author contributions: All authors contributed equally to the manuscript. S. Tanaka is the article guarantor.

Financial disclosure: None to report.

Informed consent was obtained for this case report.
